# When xylarium and herbarium meet: linking Tervuren xylarium wood samples with their herbarium specimens at Meise Botanic Garden

**DOI:** 10.3897/BDJ.9.e62329

**Published:** 2021-03-31

**Authors:** Samuel Vanden Abeele, Hans Beeckman, Tom De Mil, Cecile De Troyer, Victor Deklerck, Henry Engledow, Wannes Hubau, Piet Stoffelen, Steven B Janssens

**Affiliations:** 1 Meise Botanic Garden, Meise, Belgium Meise Botanic Garden Meise Belgium; 2 School of Integrative Plant Science, Section of Plant Biology and the L.H. Bailey Hortorium, Cornell University, Ithaca, United States of America School of Integrative Plant Science, Section of Plant Biology and the L.H. Bailey Hortorium, Cornell University Ithaca United States of America; 3 Service of Wood Biology, Royal Museum for Central Africa, Tervuren, Belgium Service of Wood Biology, Royal Museum for Central Africa Tervuren Belgium; 4 UGent-Woodlab, Gent, Belgium UGent-Woodlab Gent Belgium; 5 Department of Chemistry, SUNY Albany, Albany, United States of America Department of Chemistry, SUNY Albany Albany United States of America

**Keywords:** Central Africa, databasing, herbarium, Meise Botanic Garden, Tervuren Wood Collection, Royal Museum for Central Africa, wood specimens, xylarium

## Abstract

**Background:**

The current data paper aims to interlink the African plant collections of the Meise Botanic Garden Herbarium (BR) and the Royal Museum for Central Africa Xylarium (Tw). Complementing both collections strengthens the reference value of each institutional collection, as more complete metadata are made available and it enables increased quality control for the identification of wood specimens. Furthermore, the renewed connection enables the linking of available wood trait data with data on phenology, leaf morphology or even molecular information for many tree species, allowing assessments of performance of individual trees. In addition to studies at the interspecific level, comparisons at the intraspecific level become possible, which could lead to important new insights into resilience to and impact of global change, as well as biodiversity conservation or forest management of Central African forest ecosystems.

**New information:**

By interlinking the Tervuren Xylarium Wood database with the recently digitised herbarium of Meise Botanic Garden, we were able to establish a link between 6,621 xylarium and 9,641 herbarium records for 6,953 plant specimens. Both institutional databases were complemented with reliable specimen metadata. The Tervuren xylarium now profits from taxonomic revisions made by botanists at Meise Botanic Garden and a list of phenotypical features for woody African species can be extended with wood anatomical descriptors. New metadata from the Tw xylarium records were used to add the country of collection to 50 linked BR herbarium specimens for which this information was missing. Furthermore, metadata available from the labels on digitised BR herbarium specimens was used to update Tw xylarium records with the date of collection (817 records), collection locality (698 records), coordinates (482 records) and altitude (817 records). In conclusion, we created a reference database with reliable botanic identities which can be used in a range of studies, such as modelling analyses, community assessments or trait analyses, all framed in a spatiotemporal context. Furthermore, the linked collections hold historical reference data and specimens that can be studied in the context of global changes.

## Introduction

Tropical rainforests, occurring on four continents along the equator, are amongst the most complex ecosystems and are characterszed by the highest levels of terrestrial biodiversity on earth (Gaston 2000). While they only cover 7% land surface, they are home to more than 60% of all known species (Bradshaw et al. 2009). Furthermore, tropical forests account for almost one-third of the terrestrial gross primary production and half of the carbon stored in terrestrial biomass (Hubau et al. 2020). The Central African rainforest is the second largest rainforest block on earth and hosts more than 10,000 vascular plant species, of which approximately 4,200 are endemic, with a high proportion of woody plants, representing ~ 5% of the estimated world's tropical tree flora (Droissart et al. 2018, Sosef et al. 2017). Intact African tropical forests have been a stable carbon sink for the past three decades (until 2015), sequestering 0.66 tonnes of carbon per hectare per year (Hubau et al. 2020).

Though intact tropical forests offer major storage of carbon and are key centres of biodiversity, many questions regarding the underlying dynamics and evolutionary processes shaping that remarkable diversity remain unanswered. This is especially true for the Central African rainforest, since relatively few studies have focused on the vast tropical forests in the Congo Basin. Unfortunately, conducting necessary field surveys in the tropics is becoming more difficult due to strong intensification of the rules on access and benefit sharing and the disappearance of skilled taxonomists and field botanists with proper knowledge of the complex tropical biomes (e.g. Drew 2011, Morgera et al. 2014). As a result, established plant collections (e.g. herbarium, carpotheque, xylarium) provide a valuable alternative for field work possibilities.

One such established plant collection is the African herbarium housed at Meise Botanic Garden (Meise BG, Belgium). Although the predecessor of Meise BG was established in 1826 under the Dutch administration, the institute only became internationally renowned for its collections after the Belgian government purchased private herbarium collections from plant collectors, such as Von Martius, Blume, Galeotti and Claussen (Diagre-Vanderpelen 2012). This was accomplished during the second half of the 19^th^ century, when the Garden was managed by the famous rose specialist François Crépin. At the end of the 19^th^ century, the interest of Meise Botanic Garden shifted to Central Africa when the territory of present-day Democratic Republic of the Congo (DR Congo) was allocated to King Leopold II at the Convention of Berlin. Simultaneously, the interest in Central African plants increased as there was a growing ambition to discover new botanical products that could be exploited. This shifted the interest of study at the Botanic Garden to Central African plants. From 1888 onwards, numerous collections of Central African flora were made, with a sampling peak between 1920 and 1960. Renowned collectors who made considerable contributions to the African herbarium of the Garden were *inter alia* Déwevre, Gillet, Bequaert, Leonard, Donis, Vermoesen, Lebrun and Louis. The latter collected over 15,000 plant specimens, mostly woody species, for which wood specimens were also collected and stored at the Tervuren xylarium (see next paragraph). Due to the strong collecting efforts during that period, with the aim of studying the Central African flora, it is estimated that about 550,000 specimens were collected and deposited in the herbarium of Meise BG. This represents more than 80% of all specimens ever collected in DR Congo, Rwanda and Burundi. Overall, the African herbarium at Meise BG contains more than 950,000 herbarium sheets (www.botanicalcollections.be).

Another well-established plant collection is the Tervuren Wood Collection (xylarium) of the Royal Museum for Central Africa (RMCA, Belgium), which was founded in 1898. The reasons for establishing such an important wood collection were to connect with overseas territories, to demonstrate the importance of tropical timber for economical purposes and to improve the development of the young Belgian kingdom. Later, in the first half of the 20^th^ century, the economical aspect of wood was complemented with a scientific component. As such, the original main focus on tropical African trees with a clear commercial value (e.g. *Pterocarpus soyauxii* Taub., Khaya spp., *Nauclea diderrichii* (De Wild. & T. Durand) Merr., *Milicia excelsa* (Welw.) C.C. Berg) shifted towards the collection of wood samples in a broader scientific context. Creating herbarium vouchers to newly-collected wood samples allowed comparative wood anatomy with reliable botanical identification, which could be used in the timber trade, art history, paleoecology and archaeology. From the end of the 20th century onwards, collections of stem discs and pith-to-bark samples have been established to enable dendrochronological and forest ecological projects. As the xylarium was steadily growing and becoming more renowned worldwide, wood specimens from other continents were also incorporated into the collection. Since the 1950s, the Tervuren xylarium is considered as the governmental wood collection of Belgium (Beeckman 2003, Beeckman 2007). The xylarium has become the scientific reference collection in Belgium for wood specimens (ca. 81,000 from 13,533 species) with accompanying microtomic sections (ca. 20,500 sets of thin sections in the three principal directions) and microscope images (ca. 17,500) for wood anatomical research (Beeckman 2007, Deklerck 2019, http://xylarium.africamuseum.be).

From a global perspective, the Tervuren xylarium of the RMCA and the herbarium of Meise BG are amongst the top 5 and top 20 for their respective collections (Lynch et al. 2010, Thiers 2019). Tw and BR are the respective official acronyms for the xylarium and herbarium. Despite their enormous potential, both collections remain underexploited for scientific purposes (see Deklerck 2019 for the xylarium) and the collection databases were only recently connected with international biodiversity portals, such as GBIF.

## General description

### Purpose

The current data paper aims to interlink the African plant collections of the Meise herbarium and the Tervuren xylarium. This would strengthen the reference value of both institutions, as more complete passport data would be made available after assigning additional metadata to both the xylarium and herbarium and it would enable increased quality control on the identification of wood samples. Furthermore, the renewed connection would enable the linking of available wood trait data with data on phenology, leaf morphology or even molecular information for many tree species, allowing assessments of performance of individual trees. In addition to studies at the interspecific level, comparisons at the intraspecific level would become possible, which could lead to important new insights into resilience to and impact of global change, as well as biodiversity conservation or forest management of Central African forest ecosystems.

## Project description

### Title

Interdisciplinary exploitation of the federal Herbarium and Xylarium for tropical forest management

Acronym: HERBAXYLAREDD

### Funding

Belspo-BRAIN; axis 4 (BR/143/A3/HERBAXYLAREDD)

## Sampling methods

### Study extent

The presented dataset is based on the records for African wood specimens from the Tervuren xylarium database (available at http://xylarium.africamuseum.be) and the corresponding herbarium specimens from the Meise Botanic Garden database (available at www.botanicalcollections.be).

### Sampling description

For the herbarium, data and specimens were digitised between 2000 and 2015 by Meise BG staff, with their own funding and with the support of the Mellon Foundation, Belspo and GBiF. In 2015, the mass digitisation project "DOE!" (Digitale Ontsluiting Erfgoedcollecties - Digital Access to Cultural Heritage Collections) was funded by the Flemish Government and this allowed the digitisation of all the herbarium sheets of the African herbarium to be completed by the end of 2018. The Tervuren Xylarium Wood database was recently updated and completed with information from historical archives, thereby creating an optimised database which could be compared to the BR herbarium metadata maintained in BG-Base (Engledow et al. 2018).

By interlinking the Tervuren Xylarium Wood database with the recently-digitised herbarium of Meise Botanic Garden, we were able to match 6,953 xylarium and 10,056 herbarium records for 6,953 plant specimens (Suppl. material 1). This difference in amount of matching records is due to the fact that each herbarium sheet receives a unique herbarium number (even duplicate specimens from the same collecting event), while xylarium specimens (e.g. blocs, sections, discs) from the same individual plant are grouped under one unique Tw-number, possibly followed by an underscore with an additional number to label multiple specimens from the same organism. Furthermore, 214 of the 10,056 linked herbarium records are duplicate specimens stored at other herbaria (e.g. Yangambi, Luki, Kisangani). The complete unfiltered list also includes matched records for which discrepancies in taxon identification were observed (e.g. assigned to a different plant family, epitheton error: same genus, but different species name), as well as indeterminate specimens in either database.

### Quality control

To increase the quality and reliability of the data, we (temporarily) discarded matched records with discrepancies, i.e. (i) if both species and genus identification were different (278 records), (ii) if specimens were unidentified in the Meise BG database (10 records) or (iii) in both databases (13 records) and (iv) if specimens were only classified at the plant family level in the Meise BG database (31 records). Matched records with discrepancies (indicated as 'Unfiltered' in Suppl. material 1) can be verified and updated, based on future research, such as genetic and wood anatomical studies.

After quality filtering, we obtained a reliable reference database with linked xylarium and herbarium records for 6,621 plant specimens (indicated as 'Filtered' in Suppl. material 1). Of these, 3,516 specimens could be linked via the Tw-numbers recorded on the herbarium sheets. When such Tw-numbers were absent, a comparison of three unique variables (i.e. collector name, collector number and collection country) resulted in an additional 3,105 linked specimens. The 6,621 plant specimens with a reliable link corresponded to 6,621 wood samples and 9,728 herbarium sheets.

By linking the Tw xylarium and BR herbarium specimens, both institutional databases could be enriched with reliable specimen metadata. The Tw xylarium database was recently updated from historical archives. This additional metadata from the Tw records could be used to add the country of collection to 50 linked BR herbarium specimens where the information was missing. Furthermore, metadata available from the labels on digitised BR herbarium specimens was used to update Tw xylarium records with the date of collection (817 records), collection locality (698 records), coordinates (482 records) and altitude (817 records). Furthermore, the Tervuren xylarium benefits from taxonomical revisions made by botanists at Meise BG and the list of phenotypical features for woody African species can be extended with wood anatomical descriptors.

### Step description

In order to link the wood samples of the Tervuren xylarium with their respective herbarium specimens at Meise BG, all available data from both collections were merged.

As both collections use different unique identifiers for their specimens (BR-numbers at the Meise herbarium and Tw-numbers at the Tervuren xylarium), most wood and herbarium samples could not be linked, based on those BR and Tw identifiers. However, for some herbarium samples stored at Meise BG, the Tw-number of the associated wood sample at the Tervuren xylarium was available on the herbarium labels and in the herbarium database. In those cases, the samples could be linked directly and the match was verified by comparing the scientific taxon name, collector name and collection number of each sample. Xylarium samples, for which the Tw-number was not available in the herbarium database, were linked to their corresponding herbarium record(s) by matching a combination of three variables: collector, collector number and country of collection. After creating a uniformly formatted list of these variables for the two databases, both were merged. Linked records were verified by comparing scientific species and/or family names. Missing information (in either database) was added when possible, mostly by using metadata available from the labels on digitised herbarium sheets (available at www.botanicalcollections.be). In case of outdated taxonomic names or defunct classification systems, corrections were made so that a higher number of records could be matched. If Tw-numbers were matched to more than one BR-number, the collector name, the collector number and the date of collection were compared for these multiple hits to verify whether all BR-samples truly belonged to the same individual field collecting event.

The interlinked xylarium-herbarium data were updated in the open-access herbarium database of Meise BG (www.botanicalcollections.be), as well as in the open-access Tervuren Xylarium Wood database (http://xylarium.africamuseum.be). The complete dataset of linked specimens (Suppl. material 1) was also uploaded to the open-access repository Zenodo (DOI: 10.5281/zenodo.4534448).

## Geographic coverage

### Description

The presented dataset focussed on the Tw wood and BR herbarium specimens collected on the African continent. Sorting the linked BR- and Tw-records by country of collection (Table 1) demonstrated that approximately 75% of the specimens (4,997 specimens) were collected in DR Congo. This is due to the large collecting efforts of (Belgian) researchers at the time when DR Congo was a Belgian colony and due to the bilateral connection that is still maintained between these countries. The second best represented country was Angola with 522 specimens (ca. 8%), followed by the Republic of the Congo with 229 specimens (ca. 3%).

## Taxonomic coverage

### Description

The linked specimens belonged to 168 different plant families of which the Fabaceae was best represented (ca. 15%), followed by the Rubiaceae (ca. 10%) and Euphorbiaceae (ca. 8%). It is not surprising that the Fabaceae and Rubiaceae were the most sampled families, since they are ranked third and fourth, respectively, amongst the most species-rich flowering plant families (Christenhusz and Byng 2016). Furthermore, both families consist of several species with a woody habit, ranging from shrubs and lianas to large canopy trees. The Fabaceae also comprises a large number of tree species that are used for timber production (Mbala 2003). The species with most matches between xylarium and herbarium specimens (Table 2) was *Combretum collinum* Fresen. (18 specimens), followed by *Syzygium staudtii* (Engl.) Mildbr., *Dichapetalum madagascariense* Poir. and *Alafia ludida* Stapf, each with 17 matched specimens. The top ten of best represented species were completed with *Symphonia globulifera* L.f., *Sorindeia africana* (Engl.) Van der Veken, *Maranthes glabra* (Oliv.) Prance, *Greenwayodendron suaveolens* (Engl. & Diels) Verdc., *Entandrophragma angolense* (Welw.) C.DC. and *Dialium pachyphyllum* Harms, each with 16 linked xylarium-herbarium specimens.

## Temporal coverage

### Notes

Although collecting activities started in the late 1880s and intensified gradually, the majority of specimens in this dataset was collected at the time when DR Congo was a Belgian colony, during the 1930s (ca. 36%) and 1940s (ca. 12%), with 1937 (ca. 17%), 1936 (12%) and 1948 (6%) yielding most collections (Table 3). The number of collections reached a second peak during the 1970s and 1980s, with respectively ca. 15% and 12% of specimens collected during this period, mostly because of individual collecting efforts by Deschamps and Malaisse.

## Collection data

### Collection name

Meise Botanic Garden Herbarium; Royal Museum for Central Africa Xylarium

### Collection identifier

BR; Tw

## Usage licence

### Usage licence

Creative Commons Public Domain Waiver (CC-Zero)

## Data resources

### Data package title

List of linked Tw xylarium and BR herbarium specimens

### Alternative identifiers

10.5281/zenodo.4534448

### Number of data sets

1

### Data set 1.

#### Data set name

List of linked Tw xylarium and BR herbarium specimens

#### Number of columns

9

#### Description

Linked Tw xylarium and BR herbarium specimens with associated species name, family name, collector, collection number, collection date and country of collection.

**Data set 1.** 

Column label	Column description
Quality of link	Indicates linked records with discrepancies ('Unfiltered') and without discrepancies ('Filtered') in taxon identification
Tw number	Unique identifier of the specimen at the Royal Museum for Central Africa Xylarium (Tw)
BR number	Unique identifier of the specimen at Meise Botanic Garden Herbarium (BR)
Species name	Scientific species name
Family name	Plant family name
Collector	Name of the person(s) who collected the specimen
Collection number	Number given to the specimen by the collector(s)
Collection date	Date on which the specimen was collected
Country of collection	Country of origin of the specimen

## Supplementary Material

C80A28E5-B941-500D-9C42-2372FB12745F 10.3897/BDJ.9.e62329.suppl1 Supplementary material 1 List of linked Tw xylarium and BR herbarium specimens Data type Data sheet Brief description Linked Tw xylarium and BR herbarium specimens with associated species name, family name, collector, collection number, collection date and country of collection. File: oo_508605.txt https://binary.pensoft.net/file/508605 Vanden Abeele et al.

## Figures and Tables

**Table 1.** Number of linked specimens ('Filtered') per country of collection

**Country of collection**	**Number of specimens**
Democratic Republic of the Congo	4,997
Angola	522
Republic of the Congo	229
Rwanda	182
Uganda	175
Union of the Comoros	128
Ivory Coast	121
Burundi	90
Benin	67
Madagascar	53
Cameroon	46
Liberia	7
Tanzania	2
Morocco	1
Republic of Guinea	1
*Total*	*6,621*

**Table 2.** The 10 species for which most wood and herbarium records were linked ('Filtered'), including the corresponding plant family

**Species name**	**Family name**	**Number of specimens**
*Combretum collinum* Fresen.	Combretaceae	18
*Syzygium staudtii* (Engl.) Mildbr.	Myrtaceae	17
*Dichapetalum madagascariense* Poir.	Dichapetalaceae	17
*Alafia lucida* Stapf	Apocynaceae	17
*Symphonia globulifera* L.f.	Clusiaceae	16
*Sorindeia africana* (Engl.) Van der Veken	Anacardiaceae	16
*Maranthes glabra* (Oliv.) Prance	Chrysobalanaceae	16
*Greenwayodendron suaveolens* (Engl. & Diels) Verdc.	Annonaceae	16
*Entandrophragma angolense* (Welw.) C.DC.	Meliaceae	16
*Dialium pachyphyllum* Harms	Fabaceae	16

**Table 3.** Number of linked specimens ('Filtered') per time period

**Time period**	**Number of specimens**
1910 - 1919	104
1920 - 1929	21
1930 - 1939	2,376
1940 - 1949	812
1950 - 1959	436
1960 - 1969	175
1970 - 1979	1,023
1980 - 1989	816
1990 - 1999	306
2000 - 2009	119
2010 - 2019	224

## References

[B6389089] Beeckman Hans (2003). A xylarium for the sustainable management of biodiversity: the wood collection of the Royal Museum for Central Africa. Bulletin de l'APAD.

[B6389113] Beeckman Hans (2007). Collections of the RMCA: Wood. Series 'MUSEUM - Collecties van het KMMA'.

[B6389162] Bradshaw C. J., Sodhi N. S., Brook B. W. (2009). Tropical turmoil: a biodiversity tragedy in progress. Frontiers in Ecology and the Environment.

[B6389211] Christenhusz MJ, Byng JW (2016). The number of known plants species in the world and its annual increase. Phytotaxa.

[B6389239] Deklerck V. (2019). National treasure: valorisation of the Federal Xylarium of Belgium for timber identification and wood technology.

[B6389269] Diagre-Vanderpelen D (2012). Le Jardin botanique de Bruxelles 1826-1912: Reflet de la Belgique, enfant de l'Afrique.

[B6389277] Drew L. W. (2011). Are we losing the science of taxonomy? As need grows, numbers and training are failing to keep up. BioScience.

[B6389286] Droissart Vincent, Dauby Gilles, Hardy O. J., Deblauwe Vincent, Harris D. J., Janssens S. B., Mackinder B. A., BlachOvergaard Anne, Sonk Bonaventure, Sosef M. S.M., Stvart Tariq, Svenning JensChristian, Wieringa J. J., Couvreur T. L.P. (2018). Beyond trees: Biogeographical regionalization of tropical Africa. J Biogeogr.

[B6389316] Engledow H., De Smedt S., Groom Q., Bogaerts A., Stoffelen P., Sosef M., Van Wambeke P. (2018). Managing a mass digitization project at Meise Botanic Garden: From start to finish. Biodiversity Information Science and Standards.

[B6389343] Gaston K. (2000). Global patterns in biodiversity. Nature.

[B6389370] Hubau W., Lewis S. L., Phillips O. L. (2020). Asynchronous carbon sink saturation in African and Amazonian tropical forests. Nature.

[B6389387] Lynch Anna H., Gasson Peter E., Lens Frederic [Updated: March 2016] Index Xylariorum 4.1. http://www.iawa-website.org/.

[B6389408] Mbala S. M. (2003). Situation des ressources genetiques forestieres de la Republique democratique du Congo.

[B6389416] Morgera E., Tsioumani A., Buck M. (2014). Unraveling the Nagoya Protocol.

[B6389537] Sosef M. S.M., Dauby G., Blach-Overgaard A. (2017). Exploring the floristic diversity of tropical Africa. BMC Biology.

[B6389557] Thiers B. Index Herbariorum: A global directory of public herbaria and associated staff. http://sweetgum.nybg.org/science/ih/.

